# *BIRC6* Is Associated with Vulnerability of Carotid Atherosclerotic Plaque

**DOI:** 10.3390/ijms21249387

**Published:** 2020-12-09

**Authors:** Iraide Alloza, Andrea Salegi, Jorge Mena, Raquel Tulloch Navarro, César Martin, Patricia Aspichueta, Lucía Martínez Salazar, Jon Uriarte Carpio, Patricia De-la-Hera Cagigal, Reyes Vega, Juan Carlos Triviño, Maria del Mar Freijo, Koen Vandenbroeck

**Affiliations:** 1 Inflammation & Biomarkers Group, Biocruces Bizkaia Health Research Institute, 48903 Barakaldo, Spain; iraide.allozamoral@osakidetza.eus (I.A.); andreasalegi@kaixo.com (A.S.); jorgemena65@outlook.com (J.M.); raquel.tulloch@gmail.com (R.T.N.); 2 Department of Biochemistry and Molecular Biology, University of the Basque Country (UPV/EHU), 48940 Leioa, Spain; 3 Biofisika Institute (UPV/EHU, CSIC), 48940 Leioa, Spain; cesar.martin@ehu.eus; 4 Department of Physiology, University of the Basque Country (UPV/EHU), 48940 Leioa, Spain; patricia.aspichueta@ehu.eus; 5 Biocruces Bizkaia Health Research Institute, 48903 Barakaldo, Spain; 6 Department of Laboratory Medicine, Osakidetza, Bilbao-Basurto IHO, Basurto University Hospital, 48013 Bilbao, Spain; lucia.martinezsalazar@osakitdetza.eus (L.M.S.); jon.uriartecarpio@osakidetza.eus (J.U.C.); patricia.delaheracagigal@osakidetza.eus (P.D.-l.-H.C.); 7 Neurovascular Group, Biocruces Bizkaia Health Research Institute, 48903 Barakaldo, Spain; mariareyes.vegamanrique@osakidetza.eus (R.V.); marimar.freijoguerrero@osakidetza.eus (M.d.M.F.); 8 Sistemas Genómicos, 46980 Peterna, Spain; jc.trivino@sistemasgenomicos.com; 9 Ikerbasque, Basque Foundation for Science, 48013 Bilbao, Spain

**Keywords:** atherosclerosis, carotid plaque, *BIRC6*, autophagy, red cell distribution width, stroke

## Abstract

Carotid atherosclerotic plaque rupture can lead to cerebrovascular accident (CVA). By comparing RNA-Seq data from vascular smooth muscle cells (VSMC) extracted from carotid atheroma surgically excised from a group of asymptomatic and symptomatic subjects, we identified more than 700 genomic variants associated with symptomatology (*p* < 0.05). From these, twelve single nucleotide polymorphisms (SNPs) were selected for further validation. Comparing genotypes of a hospital-based cohort of asymptomatic with symptomatic patients, an exonic SNP in the *BIRC6* (*BRUCE*/*Apollon*) gene, rs35286811, emerged as significantly associated with CVA symptomatology (*p* = 0.002; OR = 2.24). Moreover, BIRC6 mRNA levels were significantly higher in symptomatic than asymptomatic subjects upon measurement by qPCR in excised carotid atherosclerotic tissue (*p* < 0.0001), and significantly higher in carriers of the rs35286811 risk allele (*p* < 0.0001). rs35286811 is a proxy of a GWAS SNP reported to be associated with red cell distribution width (RDW); RDW was increased in symptomatic patients (*p* < 0.03), but was not influenced by the rs35286811 genotype in our cohort. BIRC6 is a negative regulator of both apoptosis and autophagy. This work introduces *BIRC6* as a novel genetic risk factor for stroke, and identifies autophagy as a genetically regulated mechanism of carotid plaque vulnerability.

## 1. Introduction

Carotid atherosclerosis is a chronic disorder of the cervical arteries characterized by inflammation and accumulation of lipid-rich plaques in the arterial wall [1,2]. While plaque enlargement and ensuing stenosis can limit blood flow to the brain, plaque rupture can cause abrupt vascular occlusion and is a major cause of ischemic stroke. Stroke is the second leading cause of death and of disability-adjusted life years worldwide [3]. It is a complex neurologic condition of which individual risk is determined via the interplay of genetic, epigenetic, and environmental/lifestyle factors [4]. Since ischemic stroke continues to challenge modern medicine by posing both diagnostic and therapeutic difficulties, widespread efforts are being undertaken to identify biomarkers for the prediction of stroke risk in clinical practice using integrated multi-omics approaches [5]. Of these approaches, genome-wide association studies (GWAS) have already facilitated the identification of genetic risk factors associated with stroke and stroke subtypes [6,7]. A recent multi-ancestry GWAS in ischemic stroke identified 32 genome-wide significant loci, 22 of which had not been reported before [8]. Several of these individual genomic stroke risk variants are associated with related vascular traits or intermediate phenotypes for stroke including blood pressure, coronary artery disease, atrial fibrillation, carotid intima media thickness (cIMT), or carotid plaque [8]. cIMT and carotid plaque are correlated processes, but the latter constitutes a stronger predictor than cIMT for first-ever ischemic stroke [9]. Family-based studies have provided evidence for moderate heritability of cIMT and carotid plaque, and common genomic variants associated with either of both have been identified [10,11,12].

Though the basic molecular mechanisms of carotid plaque are increasingly understood [13], the molecular players involved in the transition of a stable to unstable plaque need further clarification. Plaques presenting as nonobstructive and silent arterial lesions that suddenly turn obstructive and symptomatic are considered vulnerable or unstable [14]. A combination of a fibrous cap and large lipid core, known as thin cap fibroatheroma, is a hallmark of such plaque [15]. Histological examination comparing carotid plaques removed from symptomatic with those from asymptomatic patients revealed typical features associated with vulnerability of the plaque including surface ulceration, plaque rupture, thinning of fibrous cap, and more extensive infiltration of the cap by macrophages and T lymphocytes [16]. Compared to stable plaques, vulnerable plaques are susceptible to experience more rapid progression and to produce superimposed thrombosis, which ultimately leads to acute cardio- or cerebrovascular events [17]. In the context of atherosclerosis, vascular smooth muscle cells (VSMC) can dedifferentiate, proliferate, and migrate, thus contributing to plaque formation; but they can also display beneficial roles by protecting against plaque rupture through the promotion of plaque repair. These processes are accompanied by a “phenotype switch” from a differentiated contractile to dedifferentiated synthetic state [18,19]. Recently, we performed an RNA-Seq transcriptomics screen of VSMC extracted from carotid plaques from asymptomatic and symptomatic patients in order to uncover specific gene expression signatures [20]. Sixty-seven significant differentially expressed genes were identified; enrichment and network analysis revealed a senescence-like phenotype in VSMC from vulnerable carotid atheroma that differentiates it from the osteogenic-like phenotype seen in VSMC from stable carotid plaque [20].

The present study was undertaken to identify genomic variants in the original VMSC raw dataset [20] (i.e., single nucleotide polymorphisms (SNPs) and insertions/deletions) that display potential regulatory effects on expression and exhibit distinct allele distribution in carriers of stable versus vulnerable plaques. Following validation of a selection of these variants in a larger sample collection, a *cis*-quantitative trait locus (QTL) SNP in the *BIRC6* gene emerged as significantly associated with vulnerable plaque.

## 2. Results

### 2.1. Identification of RNA-Seq-Derived Single Nucleotide Polymorphisms (SNPs) and Indels Associated with Carotid Plaque Symptomatology

We identified transcribed SNPs potentially associated with vulnerable carotid atheroma starting from the raw data of our previously performed transcriptomics analysis based on RNA-Seq of RNA isolated from VSMC of seven symptomatic patients and seven asymptomatic patients [20]. Demographic and clinical characteristics of the patients are shown in Table A1 in Appendix A. A total of 1,005,934,806 reads with an average of 77,852,486 reads per sample were obtained. Reads were aligned against the human genome using the tophat2 algorithm. The percentage of mapped reads ranged from 82.2% to 87.6% per sample, indicating a high quality of sequence data. The Genome Analysis Toolkit (GATK) tool was used to call variants including SNPs or small insertions and deletions [21]. More than 700 variants associated with symptomatology identified with GATK were classified and annotated with Variant Effect Predictor (Ensembl) with a *p* value < 0.05 [22]. Classification for impact by SnpEFF showed that 1.5% of variants had high impact (e.g., frameshift, stop gain/loss, etc.) and 5% had moderate impact effect (e.g., non-synonymous coding changes, codon insertion/deletion). The vast majority of variants (93.3%) were predicted to have low (e.g., synonymous changes, etc.) or modifier variant effects (used for terms with hard to predict effects) (Table 1). As part of this analysis, novel variants in 61 genes associated with symptomatology were uncovered.

From the full list of variants, SNPs were prioritized for validation on the basis of potential *cis*-QTL effects and/or a requirement for the variant allele to be absent in one of both clinical study groups. Three variants with significant *cis*-QTL *p* value (<0.05) were identified by the SeqGen algorithm (*PDLIM4* rs9895; *ATL3* rs79429913; *TMEM167A* rs13162274) as well as one variant with a trend for significance (*BIRC6* rs35286811 *cis*-QTL *p* value of 0.07), and each of these variants also showed absence of the variant allele in either symptomatic or asymptomatic subjects.

The selected SNPs identified by GATK analysis were filtered to identify those with a population allele frequency lower than 20% and a list of 15 variants was produced. Eight of those variants were selected to fill our target number of 12 SNPs to be analyzed (Table 2).

### 2.2. Synonymous Exonic BIRC6 SNP rs35286811 Is Associated with Vulnerable Carotid Plaque

Genotyping of the selected 12 SNPs was done in a hospital-based validation cohort consisting of 301 patients who had been subjected to surgical excision of the carotid atheroma plaque, and of whom 132 were symptomatic and 169 asymptomatic (Table A2). SNP assays were run using 96.96 Integrated Fluidic Circuit (IFC) Dynamic Arrays and SNP type assays. Risk allele frequencies of the 12 SNPs in symptomatic and asymptomatic samples are represented in Table 3. All SNPs tested showed a call rate close to 100%.

**Figure 1:**
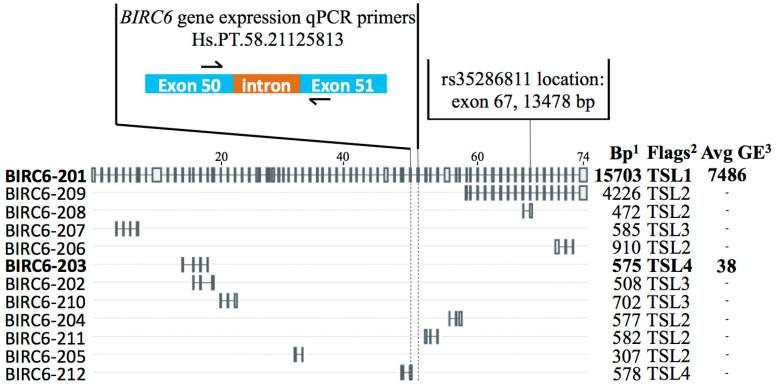
*BIRC6* gene structure. Known *BIRC6* isoforms and their exon composition are indicated based on the ENSEMBL database, with flags on the right indicating transcript support level (TSL; TSL1 is the highest score where all splice junctions of the transcript are supported by at least one non-suspect mRNA). SNP rs35286811 is located in exon 67. *BIRC6* gene expression primers (Hs.PT.58.21125813) were designed to amplify a fragment of mRNA encompassing part of exon 50 and exon 51, which is unique to the full-length isoform BIRC6-201. Average gene expression counts detected by RNA-Seq in vascular smooth muscle cells (VSMC) extracted from carotid plaque for BIRC6-201 and BIRC6-203 are indicated on the right side in bold; other *BIRC6* isoforms were not detected in these cells [20]. ^1^ Bp: base pairs. ^2^ Flags indicate the transcript support level (TSL) of each isoform. ^3^ Avg GE: average gene expression counts obtained from RNA-Seq data.

One SNP (rs35286811, *BIRC6*) demonstrated significant association with symptomatology, with the rare G allele more frequently present in symptomatic compared to asymptomatic patients (OR = 2.24, 95% C.I. = 1.27–3.93; *p* = 0.002) (Table 3). This variant is located in the 67th exon out of the 74 exons of the *BIRC6* gene and does not change the amino acid coded for (Thr4448) (Figure 1).

Additionally, two other SNPs (rs3768 in *ZNF664*; rs4726 in *SLC3A2*) showed a non-significant trend toward association (*p* < 0.1). No significant differences were found for any of the other nine analyzed SNPs. Ten out of 12 SNPs showed identical allelic trends for association with symptomatology in the RNA-Seq (Table 2) and validation (Table 3) datasets, while two SNPs (in *MGLL* and *SLK*) followed opposite trends.

### 2.3. Full-Length BIRC6 Expression Is Upregulated in Carotid Atheroma Plaques from Symptomatic Patients

**Figure 2:**
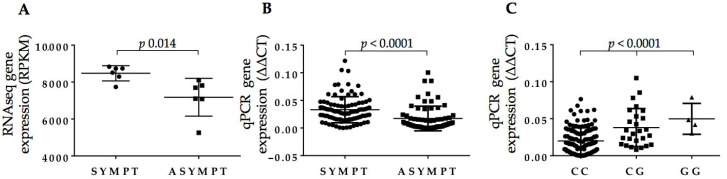
*BIRC6* gene expression levels (**A**) by RNA-Seq in the original VSMC samples extracted from 14 patients [20], and (**B**) by qPCR in extracts of carotid plaques from 70 asymptomatic and 104 symptomatic patients normalized for GAPDH and β-actin levels. In (**C**), *BIRC6* gene expression levels from (**B**) are shown classified according to rs35286811 genotype.

Upon assessment of *BIRC6* levels by RNA-Seq in the original individual 14 samples of RNA extracted from VSMC isolated from carotid plaques [20], a modest upregulation of its expression was observable in symptomatic patients with a fold-change of 1.17 and uncorrected *p* value of 0.014 (Figure 2A). In fact, two different protein-coding *BIRC6* splicing variants were detected through this RNA-Seq analysis. Of these, the full-length BIRC6-201 isoform (4857 amino acids, 530.3 kDa) generated an overall estimated average VSMC gene expression read count of 7486, while the other variant, BIRC6-203 (191 amino acids, 20.8 kDa), exhibited, at a count of 38, a ~200-fold lower expression level (Figure 1). None of the remaining *BIRC6* splice variants listed by Ensembl were detected by RNA-Seq in our setup [22]. To verify our original VSMC findings, we determined *BIRC6* expression levels in RNA extracts from lysates of full carotid plaques, which contain, in addition to VSMC, endothelial cells and infiltrated macrophages. In total, carotid plaques of 70 asymptomatic and 104 symptomatic patients were analyzed. qPCR primers were designed to amplify the *BIRC6* exon 50/51 boundary, which is unique to the full-length isoform (Figure 1 and Figure 2). Following this exercise, *BIRC6* mRNA levels appeared to be significantly higher in carotid plaques of symptomatic compared to asymptomatic patients (*p* < 0.0001; Figure 2B), which is in line with the original finding in the 14 VSMC samples (Figure 2A).

### 2.4. rs35286811 Genotype Is a cis-QTL Regulating BIRC6 Expression Levels in Carotid Plaque

As the estimated *cis*-QTL *p* value for the *BIRC6* SNP rs35286811 based on these 14 VSMC samples did not reach the significance threshold (*p* = 0.07, Table 2), we set out to verify it more robustly in an independent sample set. We assessed the effect of this SNP on *BIRC6* mRNA levels in the collection of carotid plaques stratified according to carrier genotype. As shown in Figure 2C, an allele dosage effect was observed with homozygotes for the rs35286811 G risk allele producing higher full-length *BIRC6* mRNA levels than heterozygotes, that were, in turn, higher than those of homozygotes for the ancestral C allele (*p* < 0.0001 by Kruskal–Wallis test), confirming the SNPs’ QTL effect on *BIRC6* expression in carotid plaque.

### 2.5. Linkage Disequilibrium (LD) of rs35286811 with GWAS SNPs Associated with Independent Traits Indicates Pleiotropy of the BIRC6 Locus

**Figure A1:**
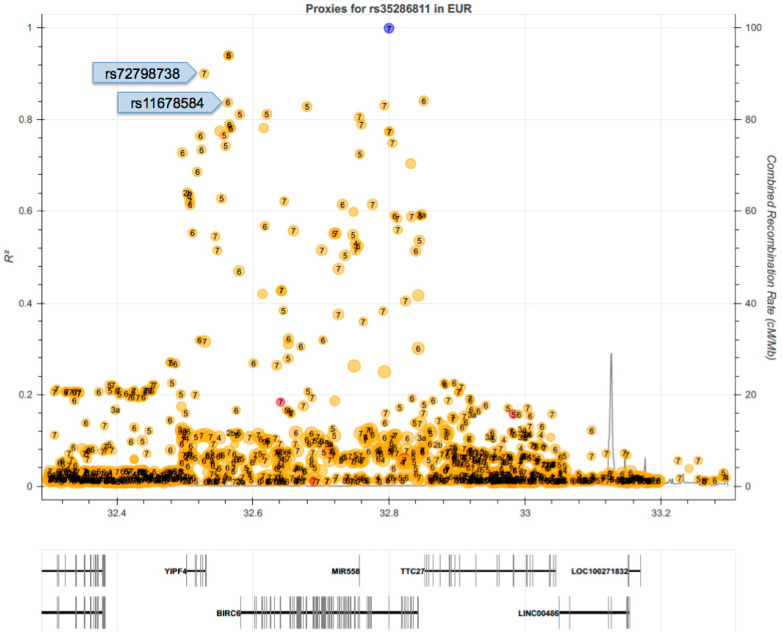
Proxies of rs35286811. Plot represents individual LD *r*^2^ values of SNPs (ochre circles) in the *BIRC6* region with rs35286811 (blue circle). GWAS-derived SNPs rs72798738 and rs11678584 are indicated. Regulatory potential of individual SNPs is scored from 1 (high) to 7 (low). Genes located in this interval are depicted in the bottom part of the figure. Image information was produced by means of https://ldlink.nci.nih.gov/.

We consulted the GWAS catalog to identify SNPs located in the wider *BIRC6* region previously reported to be associated with specific traits or disorders with genome-wide significance [23]. We calculated linkage disequilibrium (LD) between these SNPs and rs35286811 using Ldlink [24]. Of the 80 SNPs, two were identified exhibiting highly significant LD (*p* < 0.0001; *D’* > 0.95, *r*^2^ > 0.85) with rs35286811 (i.e., rs72798738 and rs11678584 (Table 4, Figure A1)), and for each of these, as for rs35286811, the minor allele was the risk allele. The first, rs72798738, was found to be associated with red cell distribution width (RDW), a measure of erythrocyte size distribution regarded as an index of patient fragility and vulnerability to adverse outcomes [25]. SNP rs11678584 was identified through association with the fraction of reticulocytes (i.e., immature red blood cells) of red cells, and with reticulocyte count [26], and it emerged also independently from a GWAS meta-analysis on chronotype (i.e., the circadian preference which determines an individual’s proclivity for earlier or later sleep timing) [27]. Thus, genetic variation at *BIRC6* tagged by rs35286811 and its proxies influences seemingly unrelated phenotypic traits.

### 2.6. RDW Is Increased in Symptomatic Patients and Is Independent from BIRC6 rs35286811 Genotype

**Figure 3:**
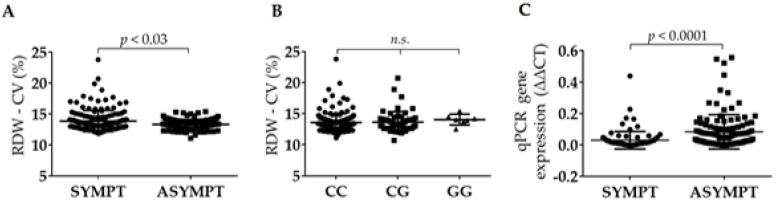
RDW-CV values in (**A**) symptomatic and asymptomatic patients, and (**B**) following stratification for rs35286811 genotype (n = 285). (**C**) *MAP1LC3B* gene expression levels by qPCR normalized for GAPDH and β-actin levels in extracts of carotid plaques from 70 asymptomatic and 104 symptomatic patients.

RDW data could be retrieved from routine hematological survey of blood samples drawn from patients shortly before surgery for removal of the carotid plaque. RDW coefficient of variation (RDW-CV) was calculated and appeared significantly higher in symptomatic compared to asymptomatic patients (*p* < 0.03; Figure 3A). In *BIRC6* genotype-stratified patients, rs35286811 did not appear to influence RDW-CV values (*p* = 0.19 by Kruskal–Wallis test; Figure 3B). mRNA levels of *MAP1LC3B*, a marker of carotid atherosclerosis [29,30], were significantly decreased in extracts from full carotid plaques from symptomatic patients (*p* < 0.0001; Figure 3C).

## 3. Discussion

Carotid plaque vulnerability is associated with risk for CVA. In this study, we identified an exonic synonymous SNP in the *BIRC6* gene of which the minor allele is significantly associated with symptomatology status in patients harboring carotid plaques [OR (95% CI) = 2.24 (1.27–3.93)]. The SNP was identified through a two-stage design involving RNA-Seq of VSMC extracted from carotid plaques from seven symptomatic and seven asymptomatic patients, followed by validation of the top-ranked SNPs in a hospital-based cohort of 169 asymptomatic and 132 symptomatic patients. *BIRC6* SNP rs35286811 genotype appeared to directly influence the expression of *BIRC6* in carotid plaque, and underlies the higher levels of BIRC6 mRNA seen in carotid plaques of the symptomatic group.

BIRC6 is a huge ubiquitin-conjugating E2 enzyme that regulates autophagosome-lysosome fusion and displays anti-apoptotic activity [31,32]. BIRC6 negatively regulates autophagy by limiting through ubiquitination the availability of MAP1LC3B, a central protein in the autophagy pathway functioning in autophagy substrate selection and autophagosome biogenesis [33,34,35,36]. In fact, autophagy is involved in multiple processes of carotid plaque formation and differentiation, and may constitute a protective mechanism to promote cell survival in the plaque [29,37]. Current research indicates that autophagy becomes dysfunctional during the progression of atherosclerosis, and that this process affects the main cellular constituents of the plaque differently [38,39]. Plaque-associated macrophages respond to autophagy impairment by undergoing apoptosis, while VSMCs engage the senescence program, with both phenomena shown to promote plaque progression [39]. High expression of MAP1LC3 has been observed by immunofluorescence staining in endothelial cells, macrophages, and VSMC in carotid plaques, but no such expression was found in the normal carotid artery [29]. *MAP1LC3B* mRNA levels were strongly decreased in carotid plaques from symptomatic compared to asymptomatic patients [30], as confirmed in this study. The work presented here suggests that the availability of bioactive MAP1LC3B may be further reduced by ubiquitination and enhanced degradation of MAP1LC3B protein brought about by genetically determined higher levels of BIRC6 in the plaques of the symptomatic group. Thus, downregulation of autophagy renders carotid plaques more vulnerable.

*BIRC6* rs35286811 is a proxy of GWAS SNPs recently found to be associated with RDW [25], reticulocyte fraction and count [26] as well as chronotype [27]. Interestingly, baseline RDW is a predictor of ischemic stroke occurrence and outcome, carotid atherosclerosis, and cerebral embolism [40,41,42]. Lappegård and colleagues showed that the link between RDW and cardiovascular morbidity and mortality could be explained by carotid atherosclerosis [43]. RDW was significantly increased in symptomatic compared to asymptomatic patients in our study, but did not vary according to the *BIRC6* rs35286811 genotype, and this indicates that carotid plaque *BIRC6* expression levels rather than RDW may explain the genotype association with CVA symptomatology in this cohort. The genome-wide association of rs72798738 with RDW was originally reported in a cohort of 445,000 individuals but OR or beta coefficient of the genetic effect size were unreported [25]; our much smaller cohort was likely underpowered to detect this trait. Considering the results of both the study of Kichaev [25] and our data combined, the QTL effect size of rs35286811 proxies on *BIRC6* mRNA levels in carotid plaque appeared much higher than that determining RDW. In addition to RDW, reticulocyte count has been reported as an independent risk factor for CVA in children with sickle cell anemia [44], and healthy sleep patterns are associated with reduced risk for cardiovascular disease and stroke [45]. Autophagy is an essential regulatory component of erythropoiesis [46], and autophagy activity is known to oscillate according to circadian rhythm [47,48]. Thus, the genetic association of *BIRC6* SNP proxies with vulnerable carotid plaque, RDW, reticulocyte fraction/count and chronotype [25,26,27] could therefore reflect shared mechanistic elements in autophagic processes.

In conclusion, we report a new SNP at the *BIRC6* locus associated with CVA symptomatology in patients harboring carotid plaques. This implies that the vulnerability of carotid plaque, and ultimately stroke risk, is partially determined by genetically regulated autophagic processes in the carotid plaque.

## 4. Materials and Methods

### 4.1. Subjects

Patients undergoing carotid endarterectomy at Basurto University Hospital from the Institute de Investigación Sanitaria (ISS) IIS Biocruces Bizkaia (Barakaldo, Spain) were recruited for selection in the current study on the bases of defined clinical parameters. Degree of stenosis was evaluated with carotid cervical Eco-Doppler ultrasound and tomographic angiography according to established criteria [49]. Symptomatic (S) patients were identified as those with >70% stenosis and presenting symptoms of transient ischemic attack or ipsilateral stroke within the past six months, while asymptomatic (A) patients were those with stenosis >80% without any presence of cerebrovascular disease over their lifetime. Only patients fulfilling all the required parameters were included in the study (i.e., 132 symptomatic and 169 asymptomatic patients). Carotid tissue samples were collected after surgery and transported immediately to the Inflammation & Biomarkers Lab for cell isolation, DNA, and RNA extraction. One day prior to surgery, blood samples were drawn from an antecubital vein into Vacutainer^®^ tubes containing EDTA as an anticoagulant (K2-EDTA 5,4 mg per tube). For blood cell counts including RDW, 3 mL of blood was drawn and analyzed within 4 h in automated blood cell counters (XN-10 and XN-20; Sysmex Europe GmbH, Norderstedt, Germany). RDW coefficient of variation (RDW-CV) was calculated by dividing the standard deviation of the mean cell size by the mean corpuscular volume (MCV) and multiplying by 100 to convert to a percentage. The study was approved by the local ethical committee (Basque Country Research Ethics Committee (CEIm-E); project identification code PI2018015; Approval date: 11 March 2019). All carotid atheroma plaques were collected from patients who had signed written informed consent. This research was performed in agreement with the principles outlined in the Declaration of Helsinki.

### 4.2. RNA-Seq Variant Detection Analysis

Genomic variants were identified in RNA-Seq data generated in our lab starting from smooth muscle cells (SMCs) purified from carotid atheroma plaques surgically excised from seven asymptomatic and seven symptomatic donors [19,20]. For variant calling, Genome Analysis Toolkit (GATK) RNA-Seq best practice guidelines were followed. GATK v3.7 was employed for SNP and indel variant discovery, to add read groups, mark duplicates, perform Split’N’Trim, reassess mapping qualities, indel realignment, base recalibration, variant calling and filtering [21]. Identified variants were functionally annotated using Variant Effect Predictor of Ensembl (VEP) [22]. QTL processing of variants with *cis* and *trans* relationship was conducted using Seqgene algorithm [50].

### 4.3. DNA and RNA Extraction from Carotid Atheroma Plaques

Genomic DNA was extracted from carotid atherosclerotic tissue with the NYZ Tissue gDNA Isolation Kit (NZYTech, Lisbon, Portugal) following the manufacturer’s instructions. DNA was quantified using the Qubit Fluorometer (Thermo Fisher, Grand Island, NY, USA) to determine the concentration and quality ratios (A260/280 and A260/230). Total RNA from carotid atherosclerotic tissue samples was extracted using the PureLink^®^ RNA Mini Kit (Thermo Fisher, Carlsbad, CA, USA) followed by DNase I treatment.

### 4.4. Selection and Genotyping of SNPs

SNP genotyping was performed using the Fluidigm 96.96 IFC Dynamic Arrays™ for Genotyping (Fluidigm, San Francisco, CA, USA). Fluidigm^®^ SNP Type™ assays were designed using the D3 assay software from Fluidigm for 12 selected SNPs [51]. Briefly, specific target amplification reaction was performed with specific primers. The diluted amplified product was loaded into the 96.96 IFC Dynamic Arrays (Fluidigm, San Francisco, CA, USA) for SNP genotyping together with amplification mixture, a ROX reference dye, and real-time master mix. Endpoint fluorescence images of the 48.48 IFC were acquired on an EP1 Fluidigm imager and the data were analyzed with Fluidigm Genotyping Analysis Software (Fluidigm, San Francisco, CA, USA).

### 4.5. Real-Time qPCR for Detection of BIRC6 and MAP1LC3B Expression in Carotid Atheroma RNA

Isolated RNA was retrotranscribed with the High-Capacity cDNA Reverse Transcription Kit (Applied Biosystem, Thermo Fisher, Grand Island, NY, USA) using 160 ng of RNA. Gene expression analysis was carried out using Fast SYBR^®^ Green Master Mix (Thermo Fisher, Carlsbad, CA, USA) on an ABI7500Fast Real-Time PCR instrument. PrimeTime qPCR primers (IDT, Leuven, Belgium) were used for amplification of *BIRC6* and *MAP1LC3B*; and GAPDH and β-actin were used as housekeeping genes for normalization. Results were analyzed using the Ct method and expressed as ∆∆Ct.

### 4.6. Statistical Analysis

RNA-Seq variants identified by GATK were analyzed for association with symptomatology using Fisher’s exact test. In order to identify differences in the distribution of genotype and allele frequencies of the 12 SNPs between symptomatic and asymptomatic patients, we used a chi-square (X^2^) test with the significance threshold set at *p* < 0.05. Statistical significance of differences in *BIRC6* gene expression levels and RDW-CV values between S and A patients were calculated with GraphPad Prism 6 (GraphPad Software, La Jolla, CA, USA) using the non-parametric Mann–Whitney U-test (one-tailed; *p* < 0.05). *BIRC6* mRNA levels and RDW-CV values were stratified according to rs35286811 genotype and compared by means of the Kruskal–Wallis test.

## 5. Patents

Title of registered industrial property: “Method to identify unstable carotid atherosclerotic plaques based on SNP identification.” Nr of application: EP19382103.0 (European application). Country of inscription: Spain. Date registered: 14/02/2019. Inventors: Iraide Alloza, Koen Vandenbroeck, María del Mar Freijo, Reyes Vega.

## Figures and Tables

**Table 1:** Summary of effect types and impact of single nucleotide polymorphisms (SNPs) identified by RNA-Seq analysis. SNPs included in this table are those associated with symptomatology with a *p* < 0.05. Effect type refers to the predicted effect the change implies. These variants can have one of four grades of biological impact: low (e.g., synonymous amino acid change), moderate (e.g., non-synonymous amino acid change), modifier (term used for difficult to predict effects; e.g., variants in 3′UTR, 5′UTR, intron, regulatory region) and high (e.g., frameshift, abolishment of splice region) impact.

Effects by Type	Counts (%)	Effects by Impact	Counts (%)
3′UTR	154 (19.5)	HIGH	12 (1.5)
5′UTR	26 (3.3)	MODERATE	40 (5)
downstream_gene	164 (20.8)	LOW	31 (4)
frameshift	11 (1.4)	MODIFIER	706 (89.5)
intron	168 (21.3)		
missense	40 (5)		
non_coding_exon	81 (10.3)		
regulatory_region	40 (5)		
splice_region	1 (0.13)		
synonymous	31 (4)		
upstream_gene	73 (9.2)		

**Table 2:** Selected candidate SNP information obtained by RNA-Seq SNP data analysis (n = 14; 7 asymptomatic and 7 symptomatic patients) ^1^ Ref. allele: allele in the reference sequence; Var. allele: variant allele observed in the sample. ^2^ Carrier counts in symptomatic (S) and asymptomatic (A) patients of the variant allele. ^3^ n.s.: not significant.

Gene	Chr	SNP Position	Ref./Var. Alleles ^1^	Variant Effect	rs Number	Carrier Counts (S/A) ^2^	*p*	*Cis*-QTL *p*
*PDLIM4*	5	132272249	G/C	synonymous	rs9895	5/0	0.021	0.03
*ATL3*	11	63625771	A/G	3_prime_UTR	rs79429913	0/5	0.021	0.04
*BIRC6*	2	32575355	C/G	synonymous	rs35286811	5/0	0.021	0.07
*TMEM167A*	5	83054823	C/T	3_prime_UTR	rs13162274	0/5	0.021	0.05
*HTT*	4	3135947	G/A	missense	rs363075	5/0	0.021	n.s. ^3^
*BDH2*	4	103998912	T/C	intron	rs6825519	5/0	0.021	n.s.
*SLK*	10	62885307	A/G	synonymous	rs10883960	5/0	0.021	n.s.
*SLC3A2*	11	55828523	C/T	synonymous	rs4726	5/0	0.021	n.s.
*NEDD4*	15	55828523	C/T	3_prime_UTR	rs2899593	5/0	0.021	n.s.
*MGLL*	3	127691190	C/G	3_prime_UTR	rs76232599	0/6	0.005	n.s.
*RMND1*	6	151405165	G/C	3_prime_UTR	rs1065310	0/6	0.005	n.s.
*ZNF664*	12	124015292	C/T	3_prime_UTR	rs3768	0/6	0.005	n.s.

**Table 3:** Association values of candidate SNPs related with symptomatology of carotid atherosclerosis in validation cohort (n = 301, 169 asymptomatic and 132 symptomatic). ^1^ RAF: risk allele frequency. ^2^ OR: odds ratio. ^3^ CI: confidence interval.

Gene	SNP	Risk Allele	RAF ^1^ Asympt	RAF Sympt	Other Allele	*p*	OR ^2^ (95% CI ^3^)
*ATL3*	rs79429913	A	0.85	0.86	G	0.72	1.09 (0.641.72)
*BDH2*	rs6825519	C	0.18	0.22	T	0.18	1.33 (0.88–1.98)
*BIRC6*	rs35286811	G	0.07	0.15	C	0.002	2.24 (1.27–3.93)
*HTT*	rs363075	A	0.09	0.10	G	0.63	1.14 (0.66–1.98)
*MGLL*	rs76232599	G	0.29	0.31	C	0.61	1.07 (0.75–1.53)
*NEDD4*	rs2899593	T	0.09	0.13	C	0.16	1.47 (0.84–2.55)
*PDLIM4*	rs9895	C	0.06	0.09	G	0.22	1.47 (0.478–2.48)
*RNMD1*	rs1065310	G	0.71	0.77	C	0.12	1.35 (0.92–1.94)
*SLC3A2*	rs4726	T	0.20	0.27	C	0.08	1.41 (0.94–2.13)
*SLK*	rs10883960	A	0.73	0.77	G	0.31	1.21 (0.83–1.77)
*TMEM167A*	rs10883960	C	0.74	0.76	T	0.41	1.12 (0.77–1.64)
*ZNF664*	rs13162274	C	0.72	0.78	T	0.06	1.45 (0.97–2.15)

**Table 4:** *BIRC6* rs35286811 proxies are associated with independent traits. ^1^ RAF: risk allele frequency in non-Finnish Europeans [28]. ^2^ Linkage disequilibrium (LD) *D’* is an indicator of allelic segregation of two genetic variants and *r*^2^ is a measure of correlation of these variants. LD values refer to European population. ^3^ Traits reported by GWAS.

SNP	Alleles (Risk Allele)	RAF ^1^	Linkage Disequilibrium (LD) ^2^			*p* for Assoc. with Trait	Trait ^3^	Ref.
			Allele Correlation	*D’*	*r* ^2^			
rs72798738	T/C (T)	0.12	rs72798738*T—rs35286811*G	0.96	0.90	7 × 10^−18^	Red cell distribution width	[25]
rs11678584	T/A (T)	0.13	rs11678584*T—rs35286811*G	0.96	0.87	2 × 10^−11^	Reticulocyte fraction of red cells	[26]
						1 × 10^−10^	Reticulocyte count	[26]
						4 × 10^−9^	Chronotype	[27]

## Abbreviations

BIRC6	Baculoviral IAP Repeat Containing 6
CI	Confidence interval
cIMT	Carotid intima media thickness
CVA	Cerebrovascular accident
LD	Linkage disequilibrium
OR	Odds ratio
QTL	Quantitative trait locus
RDW	Red cell distribution width
RDW-CV	RDW coefficient of variation
SNP	Single nucleotide polymorphism
VSMC	Vascular smooth muscle cells

## Appendix A

**Table A1:** Clinical and demographic patient data from the Biocruces Bizkaia cohort samples. Samples used for SNP identification by RNA-Seq [20].

Patient Characteristics	RNA-Seq Group	Asymp	Sympt
Number, n	14	7	7
Years	68 ± 8	68 ± 8	68 ± 8
Sex M/F, n	11/3	4/3	7/0
Treatment with statins	14	7	7
Risk Factors (%):			
Diabetes Mellitus	7 (50)	3 (43)	4 (57)
Dyslipidemia	14 (100)	7 (100)	7 (100)
Arterial hypertension	14 (100)	7 (100)	7 (100)
Tobacco	7 (50)	4 (57)	3 (43)

**Table A2:** Clinical and demographic patient data from the Biocruces Bizkaia cohort samples. Samples used for SNP validation analysis by genotyping.

Patient Characteristics	Validation Group	Asymp	Sympt
Number, n	301	169	132
Years	70 ± 15	70 ± 15	70 ± 15
Sex M/F, n	263/38	147/22	116/16
Treatment with statins	61	110	78
Risk Factors (%):			
Diabetes Mellitus	130 (43)	69 (40)	61 (41)
Dyslipidemia	209 (68)	122 (72)	87 (66)
Arterial hypertension	240 (79)	137 (81)	103 (78)
Tobacco	101 (33)	64 (37)	37 (27)
